# Lack of population differentiation patterns of previously identified putatively adaptive transposable element insertions at microgeographic scales

**DOI:** 10.1186/s13062-015-0075-4

**Published:** 2015-10-14

**Authors:** Josefa González, Jose Martínez, Wojciech Makalowski

**Affiliations:** Institute of Evolutionary Biology, CSIC-Universitat Pompeu Fabra, Barcelona, Spain; Institute of Bioinformatics, University of Muenster, Muenster, Germany

**Keywords:** Transposable elements, Evolution Canyon, Adaptation, *Drosophila melanogaster*, Environmental gradients

## Abstract

**Background:**

Transposable elements (TEs) play an important role in genome function and evolution. It has been shown that TEs are a considerable source of adaptive changes in the genome of *Drosophila melanogaster*. Specifically, footprints of selection at the DNA level, the presence of population differentiation patterns across environmental gradients, and detailed mechanistic and fitness analyses of a few candidate adaptive TEs pointed to the role of TEs in environmental adaptation. However, whether the population differentiation patterns observed at large geographic scales can be replicated at a microgeographic scale has never been assessed before.

**Results:**

In this work, we explored the population patterns of putatively adaptive TEs at a micro-spatial scale level. We compared the frequencies of TEs, previously identified as putatively adaptive and putatively neutral, in populations collected in opposite slopes of the Evolution Canyon at Mt. Carmel in Israel separated by 200 m on average. However, the differentiation patterns previously observed across large geographic distances (2000–2200 km) were not replicated at the microscale level of the Evolution Canyon populations.

**Conclusion:**

TE insertions previously associated with *D. melanogaster* environmental adaptation at a macro scale level do not play such a role at the microscale level of the Evolution Canyon populations. However, these results do not exclude a role of TEs in microgeographic adaptation because the dataset analyzed in this work is restricted to TEs identified in a single North American strain and as such is highly biased and incomplete.

**Reviewers:**

This article was reviewed by Eugene Koonin, Limsoon Wong and Fyodor Kondrashov.

## Background

Transposable elements (TE) are short repetitive sequences with the ability of moving around the genome creating new copies in the process. Although discarded for a long time as junk DNA, it is now clear that a significant fraction of TEs play an important role in genome function and evolution [1–6].

*Drosophila melanogaster* is an unrivalled model to study the role of TEs in adaptation because it has one of the best-annotated genomes, and much of the information on TE population dynamics comes from this species [7–9]. Additionally, *D. melanogaster* is a particularly good model to study environmental adaptation because it is original from tropical Africa and only recently has colonized the rest of the world [10, 11]. As such, it is likely that multiple adaptations occurred recently allowing this species to face the new environmental challenges, and some of them might be specifically related to adaptation to temperate environments [12–14]. Indeed, a recent genome-wide screen for TE-induced adaptations in *D. melanogaster* showed that TEs are a considerable source of adaptive mutations [15]. Evidence for the adaptive role of these TEs comes from the identification of footprints of selection at the DNA level, and from the presence of population differentiation patterns across environmental gradients [15, 16]. For a few of these TEs, additional evidence for their adaptive role has been obtained from detailed mechanistic and fitness analyses [17–21].

In previous works, population differentiation patterns of TEs have been investigated in populations located at the opposite ends of a cline in two different continents: in North America, Bowdoinham (Maine) and Ft. Pierce (Florida) separated by 2000 km, and in Australia, Innisfail (Queensland) and Yering Station (Victoria) separated by 2200 km. Interestingly, some of the putatively adaptive TEs showed parallel population differentiation patterns in the two continents. For some of them, a correlation between the TE frequency and environmental variables such as temperature and rainfall was observed, further suggesting a role for these TEs in local environmental adaptation [16].

While evidence for local adaptation occurring at large geographical scales is plentiful, studies addressing the presence of local adaptation over small spatial scales lagged behind [22]. It has often been assumed that gene flow will prevent population differentiation at small spatial scales [22]. However, recent works suggest that microgeographic divergence occurs across a wide range of geographic contexts and of species [22–25].

In this work, we further explored the population patterns of putatively adaptive TEs at a micro-spatial scale level. We compared the frequencies of two sets of TEs, previously identified as putatively adaptive and putatively neutral, in populations collected in opposite slopes of the Evolution Canyon (EC) at Mt. Carmel in Israel. The EC has long been used to study micro-scale patterns of evolution and several works have found differences in interslope biodiversity across life involving bacteria, fungi, plants, and animals [26]. The EC is an ideal setting to further analyze the population patterns of the identified putatively adaptive TEs at a micro-spatial level because its North Facing Slope (NFS) has a temperate climate while the South Facing Slope (SFS) has a tropical climate, and both slopes are separated by only 200 m on average [26]. Interestingly, the differentiation patterns previously observed across large geographic distances were not replicated at the micro-spatial level of the EC. These results suggest that genetic variants other than the analyzed TEs are involved in local adaptation to the different slopes of the EC.

## Results

The frequency of a set of eighteen putatively adaptive and a set of ten putatively neutral TEs, previously identified in *D. melanogaster*, was estimated in populations from opposite slopes of the Evolution Canyon (Beiles, Raz and Nevo, back-to-back submission [27]) (Table 1). Both adaptive and neutral TEs are present at low frequencies in ancestral African populations and at high frequencies in derived out-of-Africa populations. However, while putatively neutral TEs belong to families that are likely to be enriched for neutral insertions, putatively adaptive TEs belong to families that are subject to strong purifying selection and as such are more likely to be enriched for adaptive TEs [16, 28]. Therefore, the comparison between these two sets of TEs allowed us to control for the confounding effects of drift and population history on the population frequency patterns of these TEs.

**Table 1:** Frequency estimate of the previously described 18 putatively adaptive and 10 putatively neutral TEs [16] in the EC populations

			Evolutionary Canyon populations			
Flybase ID	Family	Clinal patterns^a^	NFS 5 & NSF6	SFS 1 & SFS2	Raw *p*-value	FDR *p*-value
FBti0018880	Bari1	-	0.70	0.69	0.9366	1
FBti0019056	pogo	AU08	0.84	0.79	0.5178	0.8054
FBti0019065	pogo	-	0.76	0.73	0.7272	1
FBti0019144	Rt1b	NA	0.21	0.06	0.0802	0.4489
FBti0019164	X-element	AU08	0.39	0.58	0.1293	0.5174
FBti0019170	F-element	-	0.38	0.38	0.9250	1
FBti0019372	S-element	AU08	0.25	0.37	0.2346	0.5971
FBti0019386	invader4	AU08, NA	0.48	0.32	0.1340	0.4688
FBti0019430	Doc	-	0.98	0.98	0.8514	1
FBti0019443	Rt1b	AU07, AU08	0.35	0.44	0.3695	0.7390
FBti0019624	hopper	-	0.75	0.54	0.0351	0.3278
FBti0019627	pogo	NA	0.66	0.48	0.0973	0.4542
FBti0019679	1731	-	0.89	0.87	0.7901	1
FBti0019747	F-element	-	0.15	0.21	0.4474	0.7369
FBti0020042	jockey	-	0.31	0.32	0.9035	1
FBti0020046	Doc	NA	0.21	0.43	0.0330	0.4621
FBti0020091	Rt1a	-	0.87	0.93	0.3342	0.7798
FBti0020119	S-element	AU08, NA	0.34	0.34	0.9909	0.9909
FBti0018879	BS	-	0.86	0.65	0.0252	0.7044
FBti0019079	BS	NA	0.00	0.08	0.3913	0.6847
FBti0019133	BS	-	0.69	0.89	0.0540	0.3781
FBti0019165	BS	-	0.43	0.58	0.1431	0.4006
FBti0019604	BS	-	0.33	0.34	0.8807	1
FBti0019771	1360	NA	0.40	0.40	0.9683	1
FBti0020056	BS	-	0.03	0.07	0.3674	0.7914
FBti0020057	BS	-	0.65	0.48	0.1364	0.4242
FBti0020125	BS	NA	0.53	0.50	0.7993	0.7111
FBti0020155	1360	-	0.63	0.71	0.3810	1

*FDR* false discovery rate

^a^Clinal patterns previously described in Australian populations collected in 2007 (AU07), Australian populations collected in 2008 (AU08) and North American populations (NA)

**Fig. 1:**
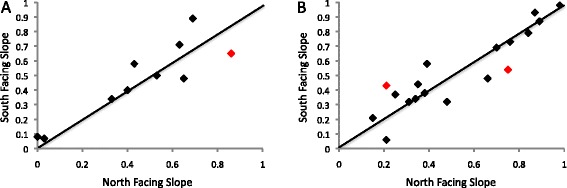
Frequencies of the putatively neutral TEs (**a**) and putatively adaptive TEs (**b**) in the North Facing Slope *vs* South Facing Slope EC populations. TEs that showed significant patterns of population differentiation before multiple testing correction are depicted in red.

We first investigated whether frequencies of neutral TEs vary between SFS populations compared to NFS populations. As expected, no significant differences were found for the putatively neutral TEs (G-test P-value = 0.156) (Fig. 1a). We then checked whether frequencies of putatively adaptive TEs were higher in the NFS population compared to SFS population, as expected if these TEs were involved in adaptation to the temperate environments. We found that overall the frequencies of putatively adaptive TEs were not significantly higher in NFS populations compared to SFS populations (G-test P-value = 0.398) (Fig. 1b).

We then investigated whether individual TE insertions showed patterns of population differentiation in the EC populations collected at opposite slopes. If we consider each TE independently, two putatively adaptive TEs, *FBti0019624* and *FBti0020046*, and one neutral TE, *FBti0018879*, showed significant population differentiation patterns (Fig. 1) (Table 1). *FBti0020046* is present at higher frequencies in SFS populations while *FBti0019624* and *FBti0018879* are present at higher frequencies in the NFS. However, none of these three TEs show significant population differentiation patterns after correcting for multiple testing (see Methods). Thus, none of the previously identified putatively adaptive TEs show population differentiation patterns at a micro-scale level.

## Discussion

In this work, we investigated whether putatively adaptive TEs play a role in microgeographic adaptation by looking for patterns of population differentiation in two EC populations separated by 200 m on average. It is important to take into account that patterns of population differentiation may simply reflect genetic structure along the populations being compared [29–31]. Thus we need to discard drift and historical processes before concluding that the population differentiation patterns are due to natural selection. To do this, we looked for population differentiation patterns in two sets of TEs previously classified as putatively adaptive and putatively neutral [16]. While drift, isolation by distance, or historical processes predict similar patterns for the neutral and adaptive TEs, selection predicts differentiation patterns only for the adaptive mutations. Our results showed that putatively adaptive TEs, previously found to show patterns of population differentiation across geographically distant clinal populations [16], do not show population differentiation patterns at a micro-scale level (Fig. 1 and Table 1).

Pavlicek et al. [32] recently demonstrated that there is substantial migration between NFS and SFS EC populations and consequently gene flow could be responsible for the lack of population differentiation that we have observed in the EC populations [33]. Although gene flow cannot be ruled out, a number of studies have shown that in the discussed system selection supersedes gene flow [34, 35]. Therefore, lack of replication of population differentiation patterns is most probably due to other factors such as to differences in the environmental conditions in temperate *vs* tropical North America and Australia populations compared to NFS *vs* SFS populations in EC. If this were the case, we would not necessarily expect the same gene/pathways to be involved in environmental adaptation in EC populations compared to temperate Australian and North American populations.

Genome-wide analyses of differentiation in North America, Australia and the EC showed that there is some overlap in the biological processes involved in environmental adaptation in these three regions [36–38]. However, the data available to date suggest that the degree of overlap is bigger between North American and Australian populations, which is consistent with our results. On the other hand, even for those genes/processes that have been identified as candidates in the three geographical regions, lack of overlap among the specific mutations responsible for environmental adaptation could be explained by their different population histories. For example, we expect a proportion of adaptive mutations to be the result of selection on standing variation, and standing variation could differ among populations [39].

## Conclusions

Although climatic differences between the slopes of Evolution Canyon are great, the distance separating the studied population is much less than in previous studies reporting TE population differentiation patterns [16]. Our results clearly demonstrate that the TE insertions associated with *D. melanogaster* adaptation at a macroscale level do not play such a role at the Evolution Canyon populations. However, these results do not exclude such a role for TEs in general. The dataset analyzed in this work is restricted to TEs identified in a single North American strain and as such is highly biased and incomplete [2, 15, 16]. *De novo* annotation of TEs in the EC populations and estimation of TE frequencies of all the annotated TEs should allow us to elucidate the role of TEs in adaptation to the different EC populations [40–43].

## Methods

### Dataset

A total of 28 TE insertions were analyzed in this study: 18 putatively adaptive TEs and 10 putatively neutral TEs that were previously described by González et al. [16]. Frequency for each of the 28 insertions was estimated in a total of 46 strains using a PCR approach as described in González et al. [15]. These 46 strains belong to four populations collected at the Evolution canyon (Lower Nahal Oren, Mount Carmel, Israel): two populations on the NFS and two populations on the SFS. To avoid the confounding effects of inversions on TE frequency estimates, strains with inversions were removed before estimating TE population frequencies. Frequency estimates for each of the four populations are given in Beiles, Raz, and Nevo (back-to-back submission) [27]. Because the TE frequencies were not significantly different within slopes, we analyzed the data of the two populations from the same slope together: NFS5 & NFS6 and SFS1 & SFS2.

### Maximum likelihood estimation of TE population frequencies

The heterogeneity in the frequencies between the NFS and SFS populations was determined using the maximum likelihood procedure described in González et al. [15]. Briefly, we compared the log-likelihoods of two models. Model H1 assumes that the frequencies in the two populations are different and estimates them using the data that come from each population separately. Model H2 assumes that the frequency of the TE is the same in both populations and estimates this frequency using the combined data from the two populations. The maximum log-likelihood under H1 and H2 are also estimated. The heterogeneity is detected when the difference between the sums of the two maximum log-likelihood values under H1 and the maximum log-likelihood value under H2 is greater than 3.84 corresponding to the 5 % critical value of the *χ*^2^ test with one degree of freedom [44].

We corrected for multiple testing following the procedure described in Benjamini and Hochberg [45].

## Reviewers’ comments

Readers should note that this manuscript was submitted and published in parallel with Beiles et al., 2015 (doi:10.1186/s13062-015-0074-5). Although submitted together, both manuscripts were reviewed independently by the same three reviewers. Some comments within the reports below may refer to Beiles et al., 2015.

### Reviewer’s report 1: Eugene Koonin. NCBI, NLM, NIH, United States of America

This work, along with the back to back paper by Beiles et al., reports tests of the hypothesis that in Drosophila, TEs previously identified as adaptive by comparison of the frequencies of widely separated populations in America and Australia could be adaptive also on the microscale, namely, in the Evolution Canyon (EC) on Mount Carmel in Israel. The conclusion is that, in contrast to the significant differences detected previously on the macroscale, on the microscale, there was no significant difference between the TE frequencies in the populations on the two slopes of the EC, and hence no evidence that the TEs from the given set are adaptive. Obviously enough, the homogenization of the TE frequencies could be attributed to the gene flow caused by migration.

To the best of my understanding, the statistical analysis that is key to this study is solid. The authors prudently note that the results only pertain to a small subset of TEs and accordingly cannot rule out adaptive roles of other TEs on the microscale.

Author’s response: We would like to thank Dr. Koonin for reviewing this manuscript.

### Reviewer’s report 2: Limsoon Wong. NUS, Singapore

There is no methodological novelty in this paper.

The contribution of this paper is purely of an observational nature. The observation was that some transposable elements (TE), previously reported (reference [16]) to be putatively involved in clinal adaptation in two different parts of the world, did not seem to be involved in clinal adaptation in an Israeli site. This does not seem important or interesting to me (Caveat: I am not an expert in evolutionary biology). Moreover, the said TE’s adaptation in reference [16] were observed from sites that are far apart (2 km), while the present one is at 200 m; so the adaptation may be non-climate-related. According to the paper, there is a parallel submission [27]. It is not clear what the difference between this and the current paper is. I should think the present observational result can be easily incorporated into the other paper. Also, reference [16] was published in PLoS Genetics, which supports reader comments. Perhaps it is more effective and more appropriate for the authors to shorten this paper into comments tagged directly to reference [16] at PLoS Genet. For these reasons, I am inclined to rejecting this paper.

Author’s response: We would like to thank Dr. Wong for reviewing this manuscript. In the first version of this manuscript we were not explicitly mentioning why investigating patterns of adaptation at macro- and micro-spatial scales is interesting. We have added a short paragraph to the introduction section to correct this.

There was a typo in our previous version of the manuscript. The distance between the previously analyzed populations is 2000 km–2200 km and not 2 km–2.2 km.

The differences between this manuscript and the back-to-back submission from Beiles et al. are in the interpretation of the results obtained. The two interpretations are too different to be included in one manuscript.

Finally, we do not agree with Dr. Wong that the results presented in this manuscript can be added as a comment to the previous publication [45]. The question that we are addressing in this manuscript is a different one, and we use a different dataset to answer it. We do believe our results should be published as an independent paper.

### Reviewer’s report 3: Fyodor Kondrashov. Center for Genomic Regulation, Spain

Review of Gonzalez et al. titled “Lack of population differentiation patterns of previously identified putatively adaptive TE insertions at microgeographic scales.” This is a simple manuscript detailing a straightforward analysis of the data contained in a back-to-back submission by Beiles and colleagues. It is, therefore, inherently linked with the other manuscript and the decision on these two manuscripts should be taken jointly. Figure 1 of the present analysis is most convincing – there do not appear to be any differences in the allele frequency of TE elements in D. melanogaster caught on the North or South slope of the Evolution Canyon. This result appears to apply to TE elements identified previously as either neutral or adaptive on much larger geographical scales. Neither do there appear to be any significant outliers with the author’s conclusion being that there is no evidence towards any of the TEs providing a selective advantage in one population versus the other. My opinion is that this analysis is solid and the manuscript best describes the data. However, I do not see how it would be possible to publish the present manuscript without the publication of the manuscript by Beiles et al., since they are the ones that have collected the data.

Author’s response: We would like to thank Dr. Kondrashov for reviewing this manuscript.

## Abbreviations

TEs: Transposable elements
EC: Evolution Canyon
NFS: North facing slope
SFS: South facing slope
